# Factors Associated With Levels of Public Engagement in Protective Behaviors During the Early COVID-19 Pandemic: Causal-Comparative Study Based on the Health Belief Model

**DOI:** 10.2196/49687

**Published:** 2023-12-19

**Authors:** Chia-Chun Tang, Hsi Chen, Shao-Yu Tsai, Wei-Wen Wu

**Affiliations:** 1 School of Nursing, National Taiwan University College of Medicine Taipei Taiwan; 2 National Taiwan University Hospital Taipei Taiwan

**Keywords:** infectious disease, protective behavior, COVID, health belief model, causal comparative, causal, protective, prevention, opinion, opinions, attitude, attitudes, COVID-19, pandemic, infection control, public safety, public health, survey, surveys

## Abstract

**Background:**

While the challenges of COVID-19 are still unfolding, the enhancement of protective behavior remains a top priority in global health care. However, current behavior-promoting strategies may be inefficient without first identifying the individuals with lower engagement in protective behavior and the associating factors.

**Objective:**

This study aimed to identify individuals with and potential contributing factors to low engagement in protective behavior during the COVID-19 pandemic.

**Methods:**

This is a causal-comparative study. A theory-based web-based survey was used to investigate individuals’ protective behavior and potential associating factors. During June 2020, the distribution of the survey was targeted to 3 areas: Taiwan, Japan, and North America. Based on the theory of the health belief model (HBM), the survey collected participants’ various perceptions toward COVID-19 and a collection of protective behaviors. In addition to the descriptive analysis, cluster analysis, ANOVA, and Fisher exact and chi-square tests were used.

**Results:**

A total of 384 responses were analyzed. More than half of the respondents lived in Taiwan, followed by Japan, then North America. The respondents were grouped into 3 clusters according to their engagement level in all protective behaviors. These 3 clusters were significantly different from each other in terms of the participants’ sex, residency, perceived barriers, self-efficacy, and cues of action.

**Conclusions:**

This study used an HBM-based questionnaire to assess protective behaviors against COVID-19 and the associated factors across multiple countries. The findings indicate significant differences in various HBM concepts among individuals with varying levels of behavioral engagement.

## Introduction

Since the emergence of COVID-19, with the first case reported in December 2019, the disease has spread globally and was declared a pandemic by the World Health Organization (WHO) in March 2020. Thereafter, the pandemic has become a series of COVID-19 waves that demonstrated different trends among regions. For example, while daily new cases reached more than 100 cases per million people in the United States in June 2020, Japan and Taiwan had about 0.49 and 0.02 daily new cases per million people, respectively. The situation reversed in September 2022, when approximate daily new cases for Taiwan, Japan, and the United States were 1723, 619, and 171 cases per million people, respectively. No matter how the pandemic surges and declines, it is clear a few years later that the world is still struggling to fight the disease [1,2]. As of June 2023, the number of confirmed cases of COVID-19 exceeded 676 million globally, with a death toll of over 6 million [3]. Therefore, preventing and slowing the transmission of the disease remains important in health care worldwide.

Despite the efforts made by the authorities to educate the public regarding the disease and promote protective behaviors, promoting these strategies may be inefficient. The webpages of the WHO and Centers for Disease Control and Prevention of many countries all have messages containing information about the current COVID-19 situation and, most importantly, encourage the practice of protective behaviors [3-6]. However, promotion strategies regarding protective measures, based on how they were shown on the government or authority websites and in publications, were mostly knowledge-based and did not deliver specific messages to at-risk groups. Such general approaches may have very limited effects, as the evidence suggests that, in addition to knowledge, several other factors may affect engagement in protective measures. For example, sex, geographic regions, perceived severity and threat, worries, and trust in the information source may all influence the adoption of protective behaviors [7-11]. Thus, it is important to identify not only the individuals who have lower compliance with protective behaviors, but also the possible contributing factors. Subsequently, tailored messages that contain crucial elements for a specific population can be designed. Furthermore, the WHO stressed on its website that it is essential for everyone to realize the importance of “doing it [protective behaviors] all [3].” Therefore, rather than focusing on a single behavior, it is necessary to look at all behaviors collectively.

To untangle the association between protective behaviors and the possible factors, it may be beneficial to use a theoretical model, such as the health belief model (HBM), to organize and conceptualize this correlation. The HBM was originally developed in the 1950s by social psychologists to enhance the effectiveness of health education programs. This model proposes that individuals’ decisions to implement disease-preventive behaviors are related to perceived susceptibility, severity, benefits, barriers, and self-efficacy. The HBM has been used widely and researchers have modified it to include cues to action, as evidence suggests that these can also affect protective behaviors [12]. Several studies have used the HBM to examine the relationship between health beliefs and protective behaviors during COVID-19. A study that examined protective behavior in Morocco and India found that perceived severity and susceptibility were vital factors that affected avoidant protective behavior, such as social distancing [11]. Another study pointed out that the self-efficacy of adolescents in Iran predicted their protective behavior, which included social distancing, wearing masks, and hand hygiene [13]. While the abovementioned evidence pointed out that specific HBM factors demonstrated powerful impacts on some protective behaviors, an Ethiopian study found that a set of HBM factors, which included self-efficacy, perceived benefits, perceived barriers, and perceived susceptibility to COVID-19, were all significant predictors of adherence to protective behaviors [14]. Alternatively, findings from an international investigation suggested that perceived severity was of little importance in predicting compliance with protective behaviors [15]. In summary, even though HBM factors have been shown to influence protective behaviors during COVID-19, the results were mixed regarding which factors made significant contributions and were different across areas. Moreover, although emerging studies have addressed protective measures against COVID-19, very few studies have investigated all the desired protective measures as a group to identify individuals who were less willing to perform these protective behaviors.

This study aimed to identify individuals with low protective behavioral engagement during COVID-19 and the potential factors that contributed to the low levels of engagement. Specifically, we aimed to (1) use an HBM-based web-based survey to describe individuals’ engagement level in protective behaviors across countries and distinguish between the low and high engagement groups and (2) identify the ascription of the factors to different groups.

## Methods

### Study Design

This cross-sectional study used a causal-comparative design. This design was selected because the groups were predetermined prior to the relationships among the variables of interest being analyzed [16].

### Recruitment

Data was collected as part of a large-scale transnational survey where the web-based survey was advertised on social media (Facebook, Instagram, and Google Ads) and the responses were recorded from June 8 to June 29, 2020. Due to budget limitations, we targeted the advertisement only to Taiwan, the United States, Canada, and Japan. Participants were included if they were aged 20 years or older and able to read and understand the selected language (English, Mandarin, or Japanese). Based on the recommendation for the estimation of a sample size for comparative studies, about 59 participants were needed for the high and low engagement groups (the proportion of the 2 groups was estimated to be 10% and 30%) [17].

### Measures

**Figure 1:**
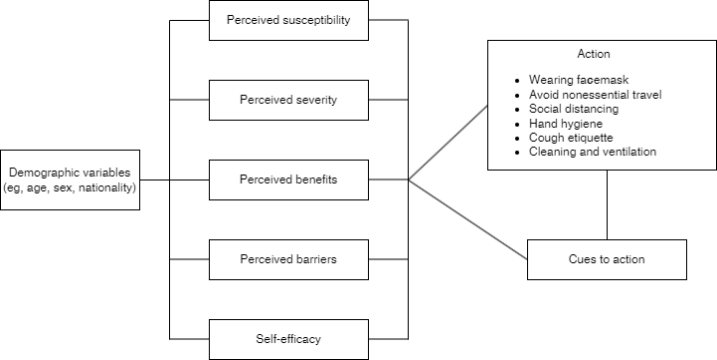
The Theoretical Model Guiding the Survey Design: Health Belief Model.

A web-based survey, designed by the investigator, was used and developed based on a literature review and the HBM (Figure 1). Details regarding the survey content and development process have been published elsewhere [18].

The survey contained 7 subscales (ie, perceived susceptibility, severity, benefits, barriers, self-efficacy, cues to action, and actions) with a total of 35 items that inquired about an individual’s perception of the pandemic and actions of protective measures. All items were rated on a 5-point Likert scale that showed the level of agreement or frequency (eg, always, sometimes, or never). Higher scores represented higher levels of agreement or more frequent adoption of behaviors. The 7 subscales were defined as follows:

- Perceived susceptibility refers to one’s belief in the likelihood of being infected by COVID-19 [19].
- Perceived severity refers to one’s feeling about the seriousness of getting the disease or of keeping it untreated [19]. Items on the perceived severity of the medical consequences on the physical and social aspects (eg, financial burden, regulation, and punishment) were formulated.
- Perceived benefits refer to an individual’s opinion on the advantages of acting on the recommended health measures [20]. Protecting oneself and others, as well as providing a sense of safety, were the most commonly indicated benefits [21].
- Perceived barriers have the following two different definitions: (1) the potential negative consequences of a particular health action that act as impediments to undertaking recommended behaviors [19] or (2) barriers that must be reduced in order to engage in the recommended behaviors [22]. We incorporated both interpretations in designing the survey.
- Self-efficacy refers to a person’s belief in their capability to execute behaviors to achieve the expected outcomes [23]. Health behavior is a series of mental and behavioral processes, which includes behavioral intention, pre-action, action, maintenance [24,25], resistance, harm reduction, coping, and recovery [26]. Factor analysis finalized 2 constructs, namely, prevention self-efficacy and maintenance self-efficacy.
- Cues to action refer to factors that might trigger an execution of the actions. We confirmed 3 constructs through factor analysis: recommendations from formal information sources (eg, government), recommendations from informal information sources (eg, friends) [27,28], and environmental cues (eg, condition of targeted places, surrounding people’s behaviors) [21].
- Action refers to preventive behaviors that can protect oneself from a COVID-19 infection. We identified and organized the proper actions suggested by the Taiwan Centers for Disease Control, the Centers for Disease Control and Prevention of the United States, the WHO, and the European Centre for Disease Prevention and Control [3-6]. There were 6 personal protective measures recommended by more than one institution that were adopted as behavioral measures, which included wearing a facemask, avoiding nonessential travel, social distancing, hand hygiene, cough etiquette, and cleaning and ventilation.

Apart from the abovementioned variables, demographic data were also included in the web-based survey. Cronbach α was .71.

### Ethical Considerations

The study was approved by the Research Ethics Committee of the National Taiwan University Hospital (202005043RINC). All participants were required to provide digital written consent before the anonymous survey began.

### Statistical Analysis

In addition to the descriptive analysis, cluster analysis was applied to group participants based on their level of engagement in all protective behaviors. The scores of the 6 behaviors were first standardized based on the *z* scores, given that the scales for these behaviors were different. Additionally, k-means clustering was used, and 3 clusters were determined using the *NbClust* package [29] in the statistical computing software R (R Foundation for Statistical Computing). ANOVA, Fisher exact tests, and chi-square tests were used to further examine the differences among the groups. Posthoc tests, Fisher least significant difference, and Bonferroni correction were applied to further clarify the directions of the aforementioned analyses. Finally, multinomial logistic regression was applied to adjust the relationships among potentially related health belief variables. A 2-sided *P*<.05 was considered statistically significant.

## Results

### Basic Information of the Participants

Among the 629 responses received, 245 (38.95%) were excluded due to duplication (n=1) or incompletion (n=244). Of the remaining 384 participants (age: mean 39.92, SD 14.65 years), 145 (37.8%) were male, 238 (62%) were female, 1 participant did not specify their sex, 106 (27.6%) were health care professionals or students, and 65 (16.9%) had chronic diseases. Nearly all participants (n=352, 91.6%) had completed a college education or higher. For the past 6 months, 258 (67.2%) participants had lived in Taiwan, 86 (22.4%) in Japan, 28 (8%) in North America, 5 (1.3%) in Europe (Switzerland, Germany, and the United Kingdom), 2 (0.52%) in Hong Kong, 1 (0.26%) in China, and 1 (0.26%) in Macau. The protective behaviors that were mostly adopted by the public were avoiding traveling abroad (n=224, 58.3%), practicing good cough etiquette (n=218, 56.8%), wearing facemasks (n=186, 48.4%), handwashing (n=179, 46.6%), cleaning and ventilating (n=128, 33.3%), and maintaining social distance (n=101, 26.3%). Table 1 displays the demographic data and the frequencies of the adopted protective behaviors.

**Table 1:** Demographic data and adopted protective behaviors (n=384).

Variables				Value
Age (years), mean (SD)				39.32 (14.65)
**Sex, n (%)**				
	Male			145 (37.8)
	Female			238 (62)
	Prefer not to answer			1 (0.3)
**Education, n (%)**				
	Primary school or lower			1 (0.3)
	Junior and senior high school			31 (8.1)
	College or university			209 (54.4)
	Graduate school			143 (37.2)
Has chronic disease, n (%)				65 (16.9)
Health care professional or student, n (%)				106 (27.6)
**Residential locations over the last 6 months, n (%)**				
	Taiwan			258 (67.2)
	Japan			86 (22.4)
	North America			31 (8.1)
	Other^a^			9 (2.3)
**Frequencies of adopted protective behavior, n (%)**				
	**Wearing facemask**			
		Never or rarely	6 (1.5)	
		Sometimes	190 (49.5)	
		Always	186 (48.4)	
	**Avoid traveling**			
		Never or rarely	9 (2.3)	
		Sometimes	148 (38.5)	
		Always	224 (58.3)	
	**Social distancing**			
		Never or rarely	25 (6.5)	
		Sometimes	257 (66.9)	
		Always	101 (26.3)	
	**Hand hygiene**			
		Never or rarely	8 (2)	
		Sometimes	196 (51)	
		Always	179 (46.6)	
	**Cough etiquette**			
		Never or rarely	5 (1.3)	
		Sometimes	159 (41.4)	
		Always	218 (56.8)	
	**Cleaning and ventilating**			
		Never or rarely	11 (2.9)	
		Sometimes	245 (63.8)	
		Always	128 (33.3)	

^a^Other locations included Switzerland, Germany, the United Kingdom, Hong Kong, China, and Macau.

### Cluster Analysis

Cluster analysis divided participants into 3 groups: those who adopted protective measures more frequently (cluster 1, high engagement; n=181, 47.1%), less frequently (cluster 2, low engagement; n=34, 8.9%), and those in-between (cluster 3, medium engagement; n=169, 44%) (Table 2). Note that since the values were standardized scores, negative values do not imply that participants did not engage in such behaviors. For instance, cluster 1 had higher standardized scores (*z* scores: 0.47334-0.67822) for all 6 behaviors than cluster 2 (*z* scores: –0.81341 to –1.65617) and cluster 3 (*z* scores: –0.29885 to –0.41468). Higher *z* scores represented more frequent adoption of protective behaviors.

**Table 2:** Final cluster centers for all participants (n=384).

Behavior	Cluster 1: high engagement (n=181), *z* score	Cluster 2: low engagement (n=34), *z* score	Cluster 3: medium engagement (n=169), *z* score	*F* test (*df*)	*P* value
Wearing facemask	0.58259	–1.61597	–0.29885	144.666 (2, 381)	<.001
Avoid traveling	.047334	–0.81341	–0.34331	52.679 (2, 381)	<.001
Social distancing	0.65691	–1.43586	–0.41468	164.140 (2, 381)	<.001
Hand hygiene	0.67822	–1.65617	–0.39318	214.035 (2, 381)	<.001
Cough etiquette	0.64384	–1.51134	–0.38550	165.072 (2, 381)	<.001
Cleaning and ventilating	0.58074	–1.21134	–0.37828	103.839 (2, 381)	<.001

### Analysis of Variance

ANOVA was used to examine whether the variables of the HBM were different among the 3 groups. The 1-way ANOVA showed significant differences in perceived barriers (*F*_2,381_=3.046, *P*=.049), self-efficacy (*F*_2,381_=23.935, *P*<.001), cues of action regarding recommendations from informal information sources (*F*_2,381_=21.152, *P*<.001), and environmental cues (*F*_2,381_=8.396, *P*<.001) (Table 3).

**Table 3:** ANOVA results between groups in terms of health belief model variables (n=384).

Dependent variables		Sum of squares (*df*)		Mean square		*F* test (*df*)		*P* value
**Perceived severity**								
	Between groups	8.732 (2)	4.366		0.685 (2, 381)		.51	
	Within groups	2429.619 (381)	6.377					
**Perceived benefit**								
	Between groups	20.492 (2)	10.246		1.829 (2, 381)		.16	
	Within groups	2133.914 (381)	5.601					
**Perceived barrier**								
	Between groups	60.615 (2)	30.308		3.046 (2, 381)		.049	
	Within groups	3790.625 (381)	9.949					
**Self-efficacy**								
	Between groups	514.435 (2)	257.218		23.935 (2, 381)		<.001	
	Within groups	4094.374 (381)	10.746					
**Cues: formal information**								
	Between groups	11.462 (2)	5.731		2.351 (2, 381)		.1	
	Within groups	928.660 (381)	2.437					
**Cues: informal information**								
	Between groups	56.089 (2)	28.044		21.152 (2, 381)		<.001	
	Within groups	505.159 (381)	1.326					
**Cues: environmental**								
	Between groups	165.485 (2)	82.743		8.396 (2, 381)		<.001	
	Within groups	3754.887 (381)	9.855					

The least significant difference posthoc test was performed to clarify the direction of the ANOVA results (Table 4). Individuals in cluster 1 perceived significantly fewer barriers than those in cluster 2 (*P*=.02). No significant differences was identified between cluster 1 and 3 or between cluster 2 and 3. For self-efficacy, cluster 1 had significantly higher scores than cluster 2 (*P*<.001) and cluster 3 (*P*<.001), while cluster 3 had significantly higher scores than cluster 2 (*P<*.001). Regarding recommendations from information sources, cluster 1 followed behavioral instructions recommended by informal sources more often than cluster 2 (*P*<.001) and cluster 3 (*P*<.001), while cluster 3 cluster followed the suggested behaviors more often than cluster 2 (*P*=.001). When making decisions about adopting protective measures, clusters 2 and 3 considered environmental cues more often than cluster 1 (*P*=.001 and *P=*.003, respectively). There was no significant difference between clusters 2 and 3 regarding the consideration of environmental cues. In order to clarify if there was an interaction between the 2 health belief variables that are significantly different among the 3 groups, perceived barriers and self-efficacy were included in the multinominal logistic regression analysis. The results showed that after controlling for perceived barriers, self-efficacy was still significantly associated with group differences (*P*<.001).

**Table 4:** Results of 1-way ANOVA and Fisher least significant difference tests examining the impact of health belief model variables on the 3 engagement levels of protective behavior during COVID-19.

Pairwise comparisons			Mean difference (SE)		95% CI		*P* value
**Perceived barrier**							
	Cluster 1^a^ vs cluster 2^b^	–1.401 (0.590)		–2.56 to –0.24		.02	
	Cluster 1 vs cluster 3^c^	–0.438 (0.337)		–1.10 to 0.23		.20	
	Cluster 2 vs cluster 3	0.963 (0.593)		–0.20 to 2.13		.11	
**Self-efficacy**							
	Cluster 1 vs cluster 2	3.944 (0.613)		2.74 to 5.15		<.001	
	Cluster 1 vs cluster 3	1.479 (0.351)		0.79 to 2.17		<.001	
	Cluster 2 vs cluster 3	–2.465 (0.616)		–3.68 to –1.25		<.001	
**Cues: informal information**							
	Cluster 1 vs cluster 2	1.248 (0.215)		0.82 to 1.67		<.001	
	Cluster 1 vs cluster 3	0.546 (0.123)		0.30 to 0.79		<.001	
	Cluster 2 vs cluster 3	–0.702 (0.216)		–1.13 to –0.28		.001	
**Cues: environmental cue**							
	Cluster 1 vs cluster 2	–2.045 (0.587)		–3.20 to –0.89		.001	
	Cluster 1 vs cluster 3	–1.019 (0.336)		–1.68 to –0.36		.003	
	Cluster 2 vs cluster 3	1.026 (0.590)		–0.13 to 2.19		.08	

^a^Cluster 1: high engagement with protective behaviors.

^b^Cluster 2: low engagement with protective behaviors.

^c^Cluster 3: medium engagement with protective behaviors.

### Categorical Data Analysis

Fisher exact and chi-square tests were used to evaluate whether categorical variables were significantly different among the clusters. The 3 clusters were significantly different based on sex (n=383, *χ*^2^_2_=8.276, *P*=.02). Bonferroni correction showed that there were significantly more men (13.8%) than women (5.9%) in cluster 2. A Fisher exact test also revealed that the clusters were significantly different based on the place of residence (*P*<.001). Bonferroni correction showed that there were fewer participants from Taiwan (37.2%) than Japan (68.6%) and North American (71%) in cluster 1, and more from Taiwan (51.6%) than Japan (27.9%) and North American (22.6%) in cluster 3.

## Discussion

### Principal Findings

This study identified individuals with different levels of engagement in protective behaviors and the significantly different characteristics among them. We categorized individuals into 3 distinct groups: high, low, and medium levels of engagement in protective behaviors. That is, a group of individuals who stuck to all protective behaviors, while another group engaged in them significantly less. While most studies focused only on the adherence to a single behavior, our study was one of the few that addressed a group of protective measures. Observing and categorizing the adherence to behaviors collectively is particularly valuable when identifying possible populations or factors contributing to gaps in outbreak prevention. These results led to the second aim of our study, which addressed the more important question of the factors associated with the different levels of behavioral engagement.

Individuals from each group were significantly different from each other in terms of both intrinsic and extrinsic factors. Intrinsic factors included sex, perceived barriers, and self-efficacy, while extrinsic factors covered cues to action and residency. For the intrinsic factors, some of our results supported the current evidence, and some demonstrated variations compared to previous studies. Similar to other studies, the results of our study confirmed that sex played an important role in behavioral engagement during a pandemic. Specifically, women were more willing to perform protective measures than men [7,10,30-34]. This suggests that men may need more health education or incentives to enhance their self-protective behaviors. Prior studies have indicated that a high level of self-efficacy was strongly associated with self-protective behaviors [9,13,14,33,35], which aligned with our finding that self-efficacy was a strong factor associated with engagement in protective behaviors, even after controlling for perceived barriers, another health belief variable that was significantly associated with group differences. Individuals with higher self-efficacy in performing protective behaviors and preventing infection are more willing to adopt protective behaviors.

Some studies conducted at the beginning of the COVID-19 pandemic showed that perceived risk, perceived susceptibility [11,14,36], perceived severity [8,9,11,35], and perceived benefits [14,30,35] predicted behavioral engagement. In contrast, our study did not find significant differences in the aforementioned variables among the groups. Our results suggested that perceived barriers were a significant contributing factor [14,30,35]. Several reasons may cause this variation, including the data collection time and location. Compared to other studies, we collected data during a relatively later period, approximately 6 months after the first reported COVID-19 case [37]. It is suspected that fear of a disease decreases when the public knows more about it. Thus, the role of fear-related concepts, such as perceived risk, perceived susceptibility, and perceived severity, in the adoption of preventive measures may not be as important as it was when COVID-19 was an unknown disease. Therefore, future studies should explore whether some intrinsic factors (eg, self-efficacy) remain fairly constant in their impact on protective behavior, while other factors may change over time or by disease status. Inconsistent results may also be related to location, as our study was the first to include a large population from Taiwan, which reported lower COVID-19 cases and deaths compared to other countries [38]. However, our results showed that Taiwanese people were a minority within the high engagement cluster. The relatively stable situation in Taiwan may not have triggered the constant urge to implement protective measures for infection prevention.

It is interesting to note that the 2 extrinsic factors, informal recommendations and environmental cues, had contrasting relationships with the adoption of protective measures. While it seems that all participants followed the recommendations from formal sources to a certain extent, our results suggested that individuals who practiced protective measures more often actually followed recommendations from informal sources more frequently. Alternatively, individuals who adopted protective measures at a medium or lower frequency were more likely to make relevant decisions based on environmental cues, such as the behaviors of surrounding people. There is a tendency for individuals with the highest adherence to protective behaviors to grasp all kinds of information and strictly follow the recommendations. However, individuals with lower adherence made their decisions more flexibly based on the changing situation. Future research should investigate whether these differences are affected by decision-making styles. For example, Scott and Bruce identified 5 distinct decision-making styles. Among these styles, the rational decision-making style is characterized as making decisions based on “a search for and logical evaluation of alternatives,” and the dependent style is “according to advice from others [39].”

This study had several limitations. First, we did not follow the behavioral changes and associated factors longitudinally. Future longitudinal studies are needed to understand the more dynamic phenomenon of the adoption of protective behaviors. Second, the web-based data collection method inevitably reached a younger population with a higher educational level. Thus, our results may not be generalizable to younger or older populations or populations with a lower educational level. The number of participants would be more representative of public opinion if the countries of origin and types of occupation were more equal in number. Specifically, while about a quarter of the study participants were health workers or students, their knowledge and training may have affected their health-related beliefs and behaviors. Future studies may explore if a health-related background can affect health beliefs and behaviors. Third, due to the lack of compensation and the length of the questionnaire, respondents’ motivation was weakened, and about 38% of the responses were incomplete. A similar phenomenon was also observed in other studies, which have shown an effective response rate of web-based surveys ranging from 10.2% to 58.6% [40,41].

### Conclusion

This study is one of the few that used an HBM-based questionnaire to survey a collection of protective behaviors against COVID-19 and the associated factors across different countries. The results identified 3 groups of people with different levels of behavioral engagement. These individuals were significantly different from each other in terms of a number of the HBM concepts, including demographics, perceived barriers, perceived self-efficacy, and cues to action. Our results are worth considering in future policy-making and research. Specifically, enhancing self-efficacy may be a powerful way to facilitate engagement in protective measures, especially since self-efficacy continuously affects individuals’ adoption of behavior regardless of the stage of the pandemic. Tailored messages targeted at men during stable but ongoing pandemic conditions are important for minimizing the possible ignorance of these protective measures. Future studies are needed to clarify whether the degree of impact of the associating factors on protective behaviors changes over time, and whether decision-making styles contribute to the engagement with protective behaviors.

## Abbreviations

HBM: health belief model
WHO: World Health Organization
